# Ischemic stroke recurrence and mortality in different imaging phenotypes of ischemic cerebrovascular disease: The SMART-MR Study

**DOI:** 10.1177/23969873231162122

**Published:** 2023-03-10

**Authors:** Carlo Lucci, Ina Rissanen, Pim A de Jong, L Jaap Kappelle, Jeroen Hendrikse, Mirjam I Geerlings

**Affiliations:** 1 Department of Radiology, University Medical Center Utrecht and Utrecht University, Utrecht, The Netherlands; 2 Julius Center for Health Sciences and Primary Care, University Medical Center Utrecht and Utrecht University, Utrecht, The Netherlands; 3 Department of Neurology, University Medical Center Utrecht and Utrecht University, Utrecht, The Netherlands; 4 Department of General Practice, Amsterdam UMC, Location University of Amsterdam, Amsterdam, The Netherlands; 5 Amsterdam Public Health, Aging & Later Life, and Personalized Medicine, Amsterdam, The Netherlands; 6 Amsterdam Neuroscience, Neurodegeneration, and Mood, Anxiety, Psychosis, Stress, and Sleep, Amsterdam, The Netherlands

**Keywords:** Stroke, mortality, imaging negative ischemia, cardiovascular disease, MRI

## Abstract

**Background:**

Diagnosis of cerebrovascular disease is based on both clinical and radiological findings, however, they do not always correlate.

**Aims:**

To investigate ischemic stroke recurrence and mortality in patients with different imaging phenotypes of ischemic cerebrovascular disease.

**Methods:**

Within the SMART-MR study, a prospective patient cohort with arterial disease, cerebrovascular diseases of participants at baseline were classified as no cerebrovascular disease (reference group, *n* = 828), symptomatic cerebrovascular disease (*n* = 204), covert vascular lesions (*n* = 156), or imaging negative ischemia (*n* = 90) based upon clinical and MRI findings. Ischemic strokes and deaths were collected at 6 month-intervals up to 17 years of follow-up. With Cox regression, relationships between phenotype and ischemic stroke recurrence, cardiovascular mortality, and non-vascular mortality were studied adjusted for age, sex, and cardiovascular risk factors.

**Results:**

Compared to reference group risk for recurrent ischemic stroke was increased not only in the symptomatic cerebrovascular disease (HR 3.9, 95% CI 2.3–6.6), but also in the covert vascular lesion (HR 2.5, 95% CI 1.3–4.8) and the imaging negative ischemia groups (HR 2.4, 95% CI 1.1–5.5). Risk for cardiovascular mortality was increased in the symptomatic cerebrovascular disease (HR 2.2, 95% CI 1.5–3.2) and covert vascular lesions groups (HR 2.3, 95% CI 1.5–3.4), while the risk was less strong but also increased in the imaging negative ischemia group (HR 1.7, 95% CI 0.9–3.0).

**Conclusions:**

People with all imaging phenotypes of cerebrovascular disease have increased risk of recurrent ischemic stroke and mortality compared to other arterial diseases. Strict preventive measures should be performed even when imaging findings or clinical symptoms are absent.

**Data access statement:**

For use of anonymized data, a reasonable request has to be made in writing to the UCC-SMART study group and the third party has to sign a confidentiality agreement.

## Introduction

Despite recent improvements in its treatment and prevention, cerebrovascular disease, mainly ischemic stroke, remains the second largest cause of death worldwide.^1,2^ Diagnosis of cerebrovascular disease is based on both clinical and radiological findings.^3^ In recent years, improvements in the magnetic resonance imaging (MRI) techniques have contributed to the diagnostics, however, clinical and radiological findings do not always correlate.^4,5^

Easier access to brain MRI has increased incidental findings of cerebrovascular lesions that had not manifested with stroke-like symptoms.^6^ The prevalence of these covert vascular lesions is estimated between ~5% and 50% depending on age group and patient characteristics.^7,8^ Covert vascular lesions are associated with poor physical and cognitive functioning and risk of recurrent stroke, dementia, and mortality.^6,9^

At the same time, it has been described that brain ischemia can be present without radiological findings.^10–14^ This clinically diagnosed brain ischemia without radiologic findings is a frequent phenomenon which usually presents as transient ischemic attacks (TIAs), but also as permanent strokes. We recently showed that individuals with imaging negative ischemia have increased brain atrophy and risk for new cortical infarcts on MRI.^15^ The clinical prognosis of patients with imaging negative ischemia remains understudied, however.

Our aim was to study association of symptomatic cerebrovascular disease, covert vascular lesions, and imaging negative ischemia with risk of recurrent clinical ischemic stroke and cardiovascular mortality during 17 years of follow-up in a prospective cohort of patients with manifest arterial disease.

## Methods

### SMART-MR study

We used data from the Second Manifestations of Arterial disease-Magnetic Resonance (SMART-MR) study. The SMART-MR study is a prospective cohort study at the UMC Utrecht, The Netherlands, aimed to examine risk factors and consequences of brain MRI abnormalities in patients with manifest arterial disease.^16^ Being one of the biggest among the seven university medical centers of the Netherlands the UMC Utrecht has a catchment area far bigger than the only province of Utrecht (population of 1.3 million) and can draw patients from the whole country. Between January 2001 and December 2005, patients newly referred to the UMC Utrecht for treatment of cerebrovascular disease, peripheral arterial disease, manifest coronary artery disease or an abdominal aortic aneurysm, and no MRI contraindications, were invited to participate. A total of 1309 patients took part in the study. Baseline assessment included a brain MRI scan, a physical examination, a carotid arteries ultrasound, laboratory tests, and questionnaires regarding vascular risk factors, medication history and general functioning. Of participants, 27 had missing MRI sequences needed for the assessment of brain infarcts and four had missing follow-up data and were excluded from the sample. The final analysis was performed on 1278 patients. The study design is similar to a previous study which noted association of ischemic imaging phenotype with the progression of brain atrophy and cerebrovascular lesions on MRI.^15^

The Medical Ethics Review Committee of UMC Utrecht approved the SMART-MR study according to the guidelines of the Declaration of Helsinki and a written informed consent was obtained by all the study participants.

### Cerebrovascular lesions on brain MRI

Cerebrovascular lesions were assessed in the subacute or chronic stage. A 1.5 Tesla whole-body machine (Gyroscan ACS-NT, Philips Medical Systems, Best, The Netherlands) was used for the brain MRI examination and included a T1-weighted (TR/TE: 235/2 ms) T2-weighted (TR/TE: 2200/11 ms), fluid-attenuating inversion recovery (FLAIR) (TR/TE/TI: 6000/100/200 ms) and Inversion Recovery (IR) (TR/TE/TI: 2900/22/410 ms) sequences. Detailed methods of data collection have been previously published.^17,18^ Cerebrovascular lesions were defined as focal hyperintensities on T2-weighted images of > 3 mm in diameter and were then characterized as lacunes or non-lacunar infarcts according to size and characteristics. Lacunes were defined as focal lesions between 3 and 15 mm according to the STRIVE criteria,^19^ whereas non-lacunar lesions were divided into large infarcts (i.e. cortical infarcts and subcortical infarcts not involving the cerebral cortex) and those located in the cerebellum or brain stem. Cerebrovascular lesions were rated visually by a trained investigator and a neuroradiologist blinded to clinical characteristics and were re-evaluated in a consensus meeting.

### History of clinical cerebrovascular disease

Clinical history of brain ischemia at baseline was based on composite scoring made of self-reported previous ischemic stroke, previous history of carotid artery operation, or a physician diagnosis at study inclusion of one among the following conditions: TIA, brain infarct, ischemic stroke, cerebral ischemia, amaurosis fugax, or retinal infarct.

### Stroke classes

Baseline stroke status of participants was classified as (1) no cerebrovascular disease (reference group), (2) symptomatic cerebrovascular disease, (3) covert vascular lesions, or (4) imaging negative ischemia according to clinical history and radiological findings described above.^15^ Patients with an history of symptomatic ischemic stroke (referred from now on as symptomatic cerebrovascular disease) were considered present if the individual had both clinical history and radiological findings. The location of an infarct did not have to agree with the symptoms for a person to be considered in the symptomatic cerebrovascular disease group. Covert ischemic vascular lesions (referred from now on as covert vascular lesions) were considered present if the individual had a radiological finding of a cerebrovascular lesion, that is, a lacune or a non-lacunar brain infarct, but denied clinical history. Imaging negative ischemia individuals had history of clinical brain ischemia based on self-reports or inclusion diagnosis but no imaging cerebrovascular lesion findings. Two authors (IR and CL) read the patient files of this group to identify subtypes of imaging negative ischemia. The patients with no cerebrovascular disease, that make up our reference group, did not have either clinical history or radiological findings of cerebrovascular disease, but they had other arterial diseases such as coronary artery disease or peripheral arterial disease.

### Covariates

At baseline, age, sex, hypertension, hypercholesterolemia, use of antiplatelet or anticoagulant medication, body mass index (BMI), diabetes, smoking habits, alcohol intake, and highest attained educational level were assessed with questionnaires and clinical examinations. Hypertension was defined as mean systolic blood pressure ⩾160 mmHg and/or mean diastolic blood pressure ⩾95 mmHg and/or use of blood-pressure lowering drugs according to the current standards of the time of data collection.^17^ The definition of hypertension was not changed to follow updated guidelines because in this sample of high-risk people with manifest arterial disease practically all participants would have hypertension according to them, which would make the adjustment pointless. Hypercholesterolemia was defined as total cholesterol >5.0 mmol/L and/or low-density lipoprotein cholesterol >3.2 mmol/L and/or use of statins. Use of anticoagulant or antiplatelet medication was defined as use of any of these medications: acetylsalicylic acid, clopidogrel, dypiridamol, fenocoprumon, or acenocumarol. BMI was calculated by dividing the body weight in kilograms by the square of the height in meters. Diabetes was defined as clinical diagnosis of diabetes and/or blood glucose ⩾7 mmol/L and/or use of oral glucose-lowering drugs or insulin. Smoking and alcohol intake were recorded via self-report questionnaires and divided in never, former, and current or recently quit. Education level was recorded via questionnaires in seven groups according to the Dutch educational system going from one being primary school to seven being an academic degree.

### Stroke recurrence and mortality

Patients received a questionnaire every 6 months via post regarding hospitalization and outpatient clinic visits to establish the recurrence of new cardiovascular events including strokes until 1st March 2018. When a cardiovascular event was reported, the patient documents were retrieved from the hospital archives and assessed in order to determine the nature of the event. Three medical doctors formed the Endpoint Committee that evaluated independently every possible new cardiovascular event.^18^ The outcomes considered for the present study were: (1) new ischemic stroke, (2) cardiovascular death, (3) non-vascular death (e.g. cancer). New ischemic stroke was classified based on hospital records as clinical stroke symptoms and impairment of at least one grade in the Modified Rankin scale regardless of the presence of infarction on MRI. TIAs were not considered as recurrent ischemic strokes. We considered as cardiovascular death any of the following events: fatal myocardial infarction, fatal ischemic stroke, fatal hemorrhagic stroke, sudden death, probable sudden death, terminal heart failure, fatal rupture of an abdominal aortic aneurysm, and other vascular causes. Non-vascular death was other causes from the ones described above. Due to limited number of events (*n* = 15), hemorrhagic strokes were not studied as outcomes.

### Statistical analysis

Of the baseline covariate data, 2.3% were missing and were imputed. The multiple imputation procedure was conducted 10 times. Baseline covariates were both imputed and used as predictors in the model except for age and sex that had no missing values. Age, sex, stroke class, logarithm of follow-up time, baseline presence of large cortical infarcts, stroke recurrence, and mortality were used only as predictors in imputation model.

Baseline characteristics are summarized with descriptive statistics: median with 95% confidence intervals (95% CI) for non-normally distributed continuous variables and counts with percentages (%) for the nominal variables. Differences in baseline characteristics were evaluated with ANOVA for continuous variables and Chi-square test for nominal variables.

Cox-proportional hazard regression was used to estimate the relationship between stroke classes (symptomatic cerebrovascular disease, covert vascular lesions, and imaging negative ischemia vs reference group) and stroke recurrence, cardiovascular mortality, and non-vascular mortality. Model 1 was adjusted for age and sex, and model 2 was additionally adjusted for educational level, hypertension, diabetes mellitus, BMI, hypercholesterolemia, smoking habits, alcohol intake, and use of anticoagulant or antiplatelet medication. Hazard Ratios (HR) with 95% CI were estimated, together with cumulative survival function curves. Duration of follow-up for stroke recurrence analysis was calculated from inclusion date until diagnosis of ischemic stroke, death, lost to follow-up, or 1st March 2018, whichever came first. Duration of follow-up for mortality analysis was calculated from inclusion date until death, lost to follow-up, or 1st March 2018.

As a first sensitivity analyses, Cox-proportional hazard regression Models 1 and 2 were repeated excluding those with only self-reported previous stroke or only history of carotid artery operation (*n* = 10) from imaging negative ischemia group. As a second sensitivity analyses, Cox-proportional hazard regression Models 1 and 2 were repeated excluding those with a history of a carotid artery operation or a carotid artery procedure during the follow up (*n* = 56) from the total sample.

IBM SPSS Statistics Version 25 for Windows (IBM Corp., Armonk, NY, USA) and R studio Version 1.3.1093 for macOS (Boston, MA, USA; URL http://www.rstudio.com) were used for the statistical analysis. In all analyses, the statistical significance was set at *p* ⩽ 0.05.

## Results

### Characteristics of the sample

Baseline characteristics for the total study sample and according to stroke classes are described in Table 1. At baseline, 204 (16%) had a symptomatic cerebrovascular disease, 156 (12%) had covert vascular lesions, 90 (7%) had imaging negative ischemia, and 828 (65%) had no previous records of cerebrovascular ischemic disease (reference group). The total follow-up time until recurrent ischemic stroke, death, or lost to follow-up was 14,875 person-years and the median follow-up time was 13.3 (IQR 5.8). Of the study sample of 1278 individuals, 120 (9.4%) were lost to follow-up during the study period.

**Table 1.** Characteristics of sample.

	Reference (*N* = 828)	Symptomatic cerebrovascular disease (*n* = 204)	Covert vascular lesions (*N* = 156)	Imaging negative ischemia (*N* = 90)	*p*-Value
Mean age, years (SD)	57.2 (9.9)	60.7 (10.2)	63.7 (8.9)	59.5 (10.9)	<0.001
Women, *n* (%)	166 (20)	41 (20)	31 (20)	22 (24)	0.790
Hypertension, *n* (%)	380 (46)	135 (66)	98 (62)	53 (59)	<0.001
Hypercholesterolemia, *n* (%)	656 (79)	155 (75)	128 (81)	70 (78)	0.495
Anticoagulant or antiplatelet medication, *n* (%)	461 (56)	127 (61)	93 (59)	64 (71)	0.025
Mean BMI, kg/m^2^ (SD)	26.9 (3.8)	26.3 (3.7)	26.9 (3.8)	27.0 (4.2)	0.248
Diabetes, *n* (%)	151 (18)	48 (23)	47 (30)	22 (24)	0.008
Smoking, *n* (%)					0.019
Never	147 (18)	24 (12)	36 (23)	21 (24)	
Former	490 (59)	124 (60)	85 (54)	41 (45)	
Current/recently quit	192 (23)	58 (28)	36 (23)	28 (31)	
Alcohol use, *n* (%)					0.703
Never	140 (17)	29 (14)	20 (13)	18 (20)	
Former	74 (9)	19 (9)	16 (10)	6 (7)	
Current/recently quit	616 (74)	158 (77)	121 (77)	66 (73)	
Mean education, classified 1–7 (SD)	3.5 (1.7)	3.6 (1.9)	3.6 (1.9)	4.3 (1.9)	0.009
Inclusion diagnosis, *n* (%)					<0.001
Cerebrovascular disease	0 (0)	168 (82)	0 (0)	68 (76)	
Peripheral arterial disease	171 (21)	13 (6)	43 (28)	7 (8)	
Coronary artery disease	539 (65)	10 (5)	70 (45)	7 (8)	
Abdominal aortic aneurysm	52 (6)	4 (2)	25 (16)	3 (3)	
Other vascular disease	66 (8)	9 (4)	18 (12)	5 (6)	
Infarct lesions on brain MRI					
Mean number of infarcts (SD)	n.a.	2.9 (2.3)	1.9 (1.6)	n.a.	<0.001
Presence of cortical infarcts	n.a.	109 (53)	41 (26)	n.a.	<0.001
Presence of lacunes	n.a.	132 (65)	105 (67)	n.a.	0.606
Presence of large subcortical infarcts	n.a.	13 (6)	1 (1)	n.a.	0.005

SD: standard deviation; *n*: number of cases.

Based on imputed data.

Individuals in the reference group had mainly coronary artery disease (65%) and peripheral arterial disease (21%) (Table 1). They were younger than individuals with any type of cerebrovascular disease and less often had hypertension, diabetes, or used anticoagulants. In all groups the median age at baseline was approximately 60 years and approximately 20% of the patients in each group were women. Cortical and large subcortical infarcts were more common in the symptomatic cerebrovascular disease group than in the covert vascular lesion group, whereas both groups had equal presence of lacunes. In imaging negative ischemia group, 9 (10%) had clinical diagnosis of ischemic stroke, 56 (62%) had clinical diagnosis of TIA, 15 (17%) had clinical diagnosis of retinal infarct or amaurosis fugax, 7 (8%) had underwent a carotid artery operation, and 3 (3%) had only self-reported stroke history.

At baseline, 30 (2.3%) persons of the total sample had a history of carotid artery operation. In the symptomatic cerebrovascular disease group this was 20 (9.8%) persons, and in the imaging negative ischemia group 10 (11.1%) persons. None of the reference group or the covert vascular lesions group had history of carotid artery operation. During the follow up 27 (2.1%) persons underwent a carotid artery procedure. In the reference group this was 11 (1.3%) persons, in the symptomatic cerebrovascular disease group 8 (3.9%), in the covert vascular lesions group 3 (1.9%), and in the imaging negative ischemia group 5 (5.6%). Of the 27 carotid artery procedures, 4 (14.8%) were endovascular and 23 (85.2%) were operative.

### Recurrent ischemic stroke

**Figure 1.**
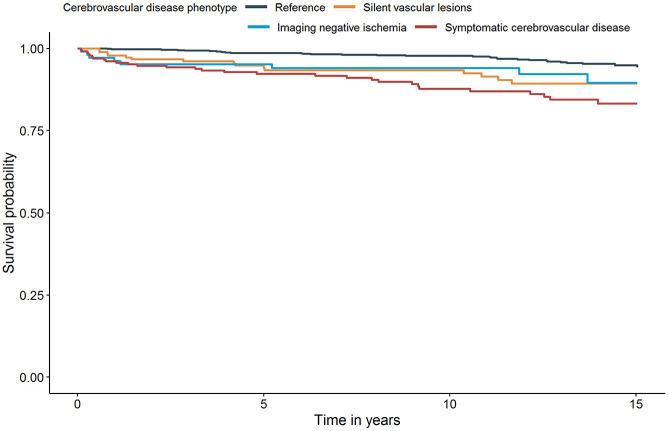
Survival curve for recurrent ischemic stroke after adjustment for age, sex, and cardiovascular risk factors (Model 2) – based on the first imputed dataset.

In total, 82/1278 (6.4%) participants had a new ischemic stroke during the follow-up resulting in a stroke incidence of 5.5/1000 person-years. Individuals with a symptomatic cerebrovascular disease at baseline had the highest incidence of recurrent ischemic stroke (13.9/1000 person-years) followed by covert vascular lesions (9.6/1000 person-years) and imaging negative ischemia (8.2/1000 person-years) (Table 2). A symptomatic cerebrovascular disease was associated with an HR of 3.9 (95% CI 2.3–6.6) compared to reference group when adjusting for age, sex, cardiovascular risk factors, and medication use (Figure 1). Individuals with covert vascular lesions (HR 2.5, 95% CI 1.3–4.8) and individuals with imaging negative ischemia (HR 2.4, 95% CI 1.1–5.5) also had an increased hazard for having a recurrent ischemic stroke after adjusting for potential confounders. Results persisted in sensitivity analyses excluding self-reported stroke (3%) or carotid artery operation (8%) from imaging negative ischemia group (data not shown), and in sensitivity analyses excluding history of a carotid artery operation (2%) or a carotid artery procedure during the follow up (2%) from the sample (data not shown).

**Table 2.** Incidence and hazard ratio of recurrent ischemic stroke and mortality in stroke classes.

	No. of events	No. of events/1000 person years	Model 1		Model 2	
			Hazard ratio	95% CI	Hazard ratio	95% CI
**Recurrent ischemic stroke**						
Reference (*n* = 828)	30	2.9	ref	/	ref	/
Symptomatic cerebrovascular disease (*n* = 204)	29	13.9	4.2	2.5–7.1	3.9	2.3–6.6
Covert vascular lesions (*n* = 156)	15	9.6	2.7	1.4–5.1	2.5	1.3–4.8
Imaging negative ischemia (*n* = 90)	8	8.2	2.5	1.1–5.6	2.4	1.1–5.5
**Vascular mortality**						
Reference (*n* = 828)	72	6.9	ref	/	ref	/
Symptomatic cerebrovascular disease (*n* = 204)	45	20.2	2.3	1.6–3.4	2.2	1.5–3.2
Covert vascular lesions (*n* = 156)	44	26.4	2.6	1.7–3.8	2.3	1.5–3.4
Imaging negative ischemia (*n* = 90)	15	14.3	1.7	1.0–3.1	1.7	0.9–3.0
**Non-vascular mortality**						
Reference (*n* = 828)	104	10.0	ref	/	ref	/
Symptomatic cerebrovascular disease (*n* = 204)	36	16.1	1.3	0.9–1.9	1.2	0.8–1.8
Covert vascular lesions (*n* = 156)	25	15.0	1.0	0.7–1.6	1.0	0.6–1.5
Imaging negative ischemia (*n* = 90)	6	5.7	0.5	0.2–1.1	0.5	0.2–1.1

Model 1: adjusted for age and sex; Model 2: adjusted for hypertension, hypercholesterolemia, use of anticoagulant or antiplatelet medication, BMI, diabetes, smoking, alcohol use, and education level; CI: confidence interval; ref: reference group.

### Mortality

**Figure 2.**
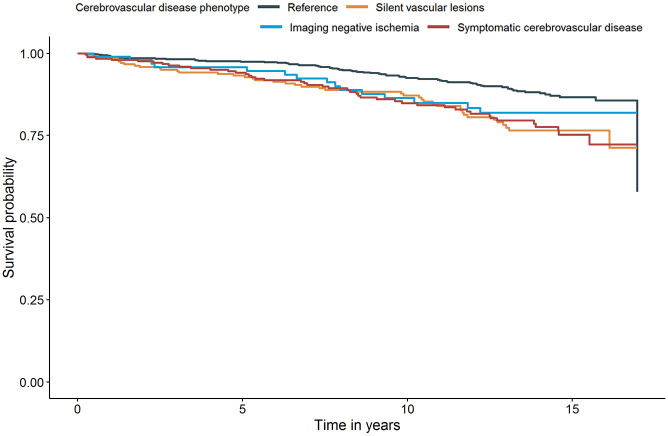
Survival curve for cardiovascular mortality after adjustment for age, sex, and cardiovascular risk factors (Model 2) – based on the first imputed dataset.

**Figure 3.**
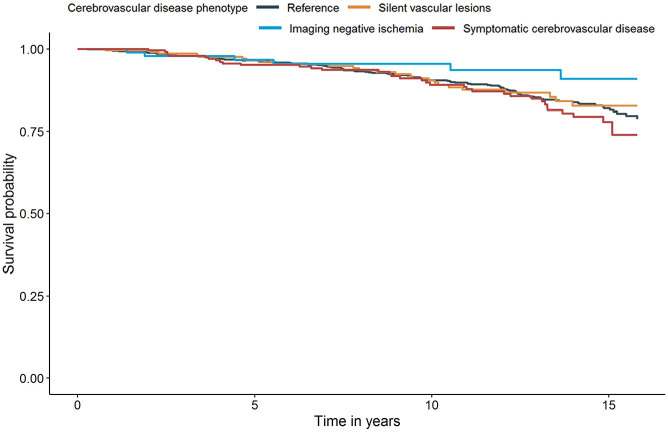
Survival curve for non-vascular mortality after adjustment for age, sex, and cardiovascular risk factors (Model 2) – based on the first imputed dataset.

In total 375/1278 (29.3%) participants died during the follow-up resulting the mortality to be 24.5/1000 person-years. Of these deaths 176 (47%) had a cardiovascular cause, 171 (46%) had a non-vascular cause and 28 (7%) had an unknown cause. Mortality rates and results of the Cox regression analysis of the associations between stroke class and cardiovascular or non-vascular mortality are shown in Table 2. Cardiovascular mortality was highest among individuals with covert vascular lesions (26.4/1000 person-years) followed by symptomatic cerebrovascular disease (20.2/1000 person-years) and imaging negative ischemia (14.3/1000 person-years). When age, sex, cardiovascular risk factors, and medication use were adjusted for, individuals with symptomatic cerebrovascular disease (HR 2.2, 95% CI 1.5–3.2) and covert vascular lesions (HR 2.3, 95% CI 1.5–3.4) had increased hazard of cardiovascular mortality compared to reference group (Figure 2). Individuals with imaging negative ischemia showed an increased hazard of cardiovascular mortality, however it lost statistical significance in model 2 (HR 1.7, 95% CI 0.9–3.0). Non-vascular mortality did not differ among groups (Figure 3). Results persisted in sensitivity analyses excluding self-reported stroke (3%) or carotid artery operation (8%) from imaging negative ischemia group (data not shown), and in sensitivity analyses excluding history of a carotid artery operation (2%) or a carotid artery procedure during the follow up (2%) from the sample (data not shown).

## Discussion

We found that not only symptomatic cerebrovascular disease but also covert vascular lesions and imaging negative ischemia, mainly TIA, are associated with increased risk for recurrent ischemic stroke and cardiovascular mortality as compared to other atherosclerotic diseases. The finding remained even when age, sex, cardiovascular risk factors, and medications were taken into account. The risk for non-vascular mortality was similar in all phenotypes of cerebrovascular disease compared to people with other atherosclerotic diseases.

Our results confirm previous knowledge that individuals with symptomatic cerebrovascular disease have two-times increased cardiovascular mortality and four-times increased risk of recurrent ischemic stroke.^20^ However, absolute rates of recurrent ischemic stroke were relatively low compared to previous studies,^21^ which may be due to treatment effects or competing risk due to increased mortality. Differently from previous research approaches, in this study from a large cohort of individuals with arterial disease we studied ischemic stroke recurrence and mortality separately in groups where either clinical history or radiological manifestations of cerebral ischemia were absent. We found that individuals with covert vascular lesions had nearly three-times increased risk of clinical ischemic stroke recurrence, which is in line with previous studies.^11^ Furthermore, their cardiovascular mortality was highest of all groups with a similar hazard ration than found in previous studies.^22^

We are among the first to report the risk of ischemic stroke recurrence and cardiovascular and non-vascular mortality among patients with imaging negative ischemia. Our study shows that these patients have increased risk for ischemic stroke recurrence and cardiovascular mortality, even when compared to people with other arterial disease and adjusted for potential confounders. Of note, the size of the risk was similar to that of patients with symptomatic cerebral ischemia or covert vascular lesions. In concordance with our findings, a previous study observed that patients with acute clinical stroke without manifest alterations on MRI had a similar prognosis with respect to the future stroke risk as patients with an imaging positive lesion.^12^ It is notable that imaging negative ischemia patients are a common entity in clinical practice,^23^ however, a heterogeneous group regarding clinical etiology, presentation, and duration of symptoms. In our sample, 62% of imaging negative ischemia group had clinical diagnosis of a TIA. Our results support the literature on importance of effective treatment of TIA and other imaging negative ischemia to prevent future subsequent ischemic stroke and death,^24^ also in chronic phase.

We previously showed that not only persons with symptomatic cerebrovascular disease but also those with covert vascular lesions or imaging negative ischemia have increased brain atrophy and risk for new infarct lesions on MRI during 12 years of follow-up compared to individuals with other arterial diseases.^15^ However, imaging negative ischemia was not associated with development of white matter hyperintensities in this study. Covert vascular lesions are linked to cerebral small vessel disease,^25^ and so is imaging negative ischemia, but in a different way. The difference between covert and symptomatic cerebrovascular disease could be explained by lesion location as subcortical lesions are more often covert.^15,26^ Furthermore, small cortical lesions are more likely than subcortical lesions to disappear completely from MRI,^27^ which may explain the manifestation as imaging negative ischemia. Overall, small vessel disease burden is a potential explanation behind the stroke recurrence and mortality risk both in covert vascular lesions and imaging negative ischemia.

Increased risk of recurrent stroke, mortality, brain atrophy, and vascular brain lesions in covert vascular lesions and imaging negative ischemia might be caused by the different treatment that patients with symptomatic and radiologically visual cerebrovascular disease receive compared to them.^6^ However, differences in use of anticoagulant or antiplatelet medications do not seem to explain the findings as they were adjusted for. Interestingly, use of these medications was similar in reference group (56%), symptomatic cerebrovascular disease (61%), and covert vascular lesions (59%), and highest in imaging negative ischemia (71%). Notably, the entire study population consisted of patients with arterial disease and the increased risk for adverse outcomes was detected when compared to people with diseases such as coronary artery disease or peripheral arterial disease. The effect of covert vascular lesions or imaging negative ischemia on adverse outcomes might thus be even larger when compared to healthy people. Our findings highlight the importance of detection and effective treatment in all forms of brain ischemia even when clinical or imaging findings are absent.

The strengths of our study include the long follow-up period of up to 17 years and the large number of individuals included that add significantly to previous research. Another strength was that only 9% of study sample were lost to follow-up. Furthermore, the cohort consisted of a large number of individuals with arterial disease and ischemic cerebrovascular disease. Hence our cohort provides real world data and produces evidence on patients that are often seen in the daily practice. Finally, the extensive information on markers of cerebrovascular pathology and cardiovascular risk factors allowed us to investigate whether the findings of this study were independent of possible confounders and comorbid arterial diseases.

Our interpretation of the results may be limited by a few factors. Although we corrected our analysis for baseline use of antiplatelet or anticoagulant medication and baseline level of cardiovascular risk factors, there might still be residual confounding. Any changes in treatments or interventions during the follow-up were not taken into account. Treatment and secondary prevention of stroke has changed considerably during the past 20 years which may affect the applicability of our findings today. Especially, advances in acute stroke treatment, better recognition of etiological and risk factors, such as extended rhythm monitoring, and the introduction of new direct oral anticoagulants are likely to influence the stroke prognosis, recurrence, and mortality.^28–30^ With better treatment and secondary prevention, the stroke recurrence and mortality rate is likely to be smaller than 20 years ago. Another limitation is the use of 1.5 Tesla MRI that might have played a role in not detecting small ischemic lesions in the imaging negative ischemia group. Furthermore, the size and vascular territory of covert and symptomatic cerebrovascular disease or the stroke etiology were not taken into account. Because the relevant location of the infarct and correlated symptoms were not considered, there might be obscureness between symptomatic cerebrovascular disease and covert vascular lesions. Fourth, brain imaging data at original stroke were not available and relevance of the infarct lesions to the symptoms were not corroborated. The symptoms and the MRI findings in the symptomatic cerebrovascular disease group may not overlap. Fifth, some symptomatic patients were identified only based on their self-report of having had a stroke, however, their number was small, and the findings persisted in sensitivity analyses excluding them from the sample. Finally, the indication of the anticoagulant or antiplatelet medication was unknown. That could be a confounding factor for stroke recurrence because the recurrence of the cardioembolic source could differ from that of atherosclerotic origin.

## Conclusions

This longitudinal study with up to 17 years of follow-up showed that risk for ischemic stroke recurrence and cardiovascular mortality are increased in all manifestations of cerebral ischemia compared to people with other arterial diseases. This highlights that a strict follow-up and preventive measures are beneficial to cerebral ischemia patients even when brain imaging findings or clinical symptoms are absent.

**Table:** The UCC-SMART Study Group.

Name	Location	Role	Contribution
FLJ Visseren, MD, PhD	University Medical Center Utrecht, Utrecht University, Utrecht, NL	Chairman; co-investigator; SMART study contributor	Responsible for data integrity; responsible for endpoint adjudication
FW Asselbergs, MD, PhD	University Medical Center Utrecht, Utrecht University, Utrecht, NL	Co-investigator; SMART study contributor	Responsible for data integrity; responsible for endpoint adjudication
HM Nathoe, MD, PhD	University Medical Center Utrecht, Utrecht University, Utrecht, NL	Co-investigator; SMART study contributor	Responsible for data integrity; responsible for endpoint adjudication
ML Bots, MD, PhD	Julius Center for Health Sciences and Primary Care, Utrecht University, Utrecht, NL	Co-investigator; SMART study contributor	Critical review; responsible for data integrity; responsible for endpoint adjudication
MI Geerlings, PhD	Julius Center for Health Sciences and Primary Care, Utrecht University, Utrecht, NL	Co-investigator; SMART study contributor	Critical review; responsible for data integrity; responsible for endpoint adjudication
MH Emmelot, MD, PhD	University Medical Center Utrecht, Utrecht University, Utrecht, NL	Co-investigator; SMART study contributor	Responsible for data integrity; responsible for endpoint adjudication
GJ de Borst, MD, PhD	University Medical Center Utrecht, Utrecht University, Utrecht, NL	Co-investigator; SMART study contributor	Critical review; responsible for data integrity; responsible for endpoint adjudication
LJ Kappelle, MD, PhD	University Medical Center Utrecht, Utrecht University, Utrecht, NL	Co-investigator; SMART study contributor	Critical review; responsible for data integrity; responsible for endpoint adjudication
T Leiner, MD, PhD	University Medical Center Utrecht, Utrecht University, Utrecht, NL	Co-investigator; SMART study contributor	Responsible for data integrity; responsible for endpoint adjudication
PA de Jong, MD, PhD	University Medical Center Utrecht, Utrecht University, Utrecht, NL	Co-investigator; SMART study contributor	Critical review; responsible for data integrity; responsible for endpoint adjudication
AT Lely, MD, PhD	University Medical Center Utrecht, Utrecht University, Utrecht, NL	Co-investigator; SMART study contributor	Responsible for data integrity; responsible for endpoint adjudication
NP van der Kaaij, MD, PhD	University Medical Center Utrecht, Utrecht University, Utrecht, NL	Co-investigator; SMART study contributor	Responsible for data integrity; responsible for endpoint adjudication
Y Ruigrok, MD, PhD	University Medical Center Utrecht, Utrecht University, Utrecht, NL	Co-investigator; SMART study contributor	Critical review; responsible for data integrity; responsible for endpoint adjudication
MC Verhaar, MD, PhD	University Medical Center Utrecht, Utrecht University, Utrecht, NL	Co-investigator; SMART study contributor	Critical review; responsible for data integrity; responsible for endpoint adjudication
J Westerink, MD, PhD	University Medical Center Utrecht, Utrecht University, Utrecht, NL	Co-investigator; SMART study contributor	Critical review; responsible for data integrity; responsible for endpoint adjudication
